# Hemorrhagic stroke following snake bite in Burkina Faso (West Africa). A case series

**DOI:** 10.1186/s40794-021-00150-6

**Published:** 2021-09-01

**Authors:** Alfred Anselme Dabilgou, Apoline Sondo, Alassane Dravé, Ismael Diallo, Julie Marie Adeline Kyelem, Christian Napon, Jean Kaboré

**Affiliations:** 1 Department of Neurology, University Hospital Yalgado Ouedraogo, 03 BP 7022, Ouagadougou, Burkina Faso; 2 Department of Neurology, Souro Sanon University Hospital Teaching Hospital, Bobo Dioulasso, Burkina Faso; 3 Infectious Diseases Department, University Hospital Yalgado Ouedraogo, Ouagadougou, Burkina Faso; 4 Department of Neurology, Regional University Hospital of Ouahigouya, Ouahigouya, Burkina Faso; 5 Department of Neurology, University Hospital of Bogodogo, Ouagadougou, Burkina Faso

**Keywords:** Snake bite, Antivenin, Hemorrhagic stroke, Burkina Faso

## Abstract

**Background:**

Snake bites remain a major medical problem in West Africa, and hemorrhagic stroke following a snakebite has emerged as a rare secondary condition. The objective of this study was to determine the neurological complications following snake bite.

**Methods:**

This study included all the cases of hemorrhagic stroke following snake bite admitted in the neurology Department of Yalgado Ouedraogo University Teaching Hospital during the period from January 1st, 2018 to December 31st 2019.

**Results:**

Three cases of hemorrhagic stroke following snake bite were included in the study. The strokes occurred 4–15 days after the snakebite. Traditional treatment was applied in two cases. Complications were significant, including local manifestations and severe anemia in 2 patients who received blood transfusion. Snake anti-venom was applied. At admission, motor deficit, conscience disorders and fever were the most frequent complaints. Patients received repeated dose of snake anti-venom was applied, antitetanus prophylaxis and antibiotherapy during hospitalization. The majority of the patients had completely recovered.

**Conclusions:**

Hemorrhagic stroke following snake bites are rare in Burkina Faso. Clinical outcome of stroke was favorable after treatment by antivenom, anti-tetanus serum and antibiotics.

## Background

Snakebites are a major medical problem among rural communities of the savanna region of West Africa, notably in Benin, Burkina-Faso, Cameroon, Ghana, Nigeria and Togo [1]. Snake venom is a complex animal poison that leads to the development of several neurologic complications. Venomous snakes can cause stroke due to either their neurotoxic or hemotoxic enzymes [2]. According to Al-Sadawi et al., ischemic strokes account for 77.1% of the cases while intracerebral hemorrhage account for 20.5% [3]. Intracerebrale hemorrhage after snake bite has been sporadically reported in different regions of the world and it is an important prognostic factor, associated with high mortality [4]. In Africa, there are few studies reporting cases of hemorrhagic stroke following snake bite studies in the literature [5, 6]. No cases have been reported in Burkina Faso despite significant reporting [7, 8]. The objective of this study was to describe the cases of hemorrhagic stroke following snake bite in a tertiary hospital in Burkina Faso.

## Methods

This study included all the cases of hemorrhagic stroke following snake bite admitted in the neurology Department of Yalgado Ouedraogo University Teaching Hospital during the period from January 1st, 2018 to December 31st 2019.

### Cases presentations

Three cases of hemorrhagic stroke following snake bite admitted snake were included in the study.

### Case 1

**Fig. 1:**
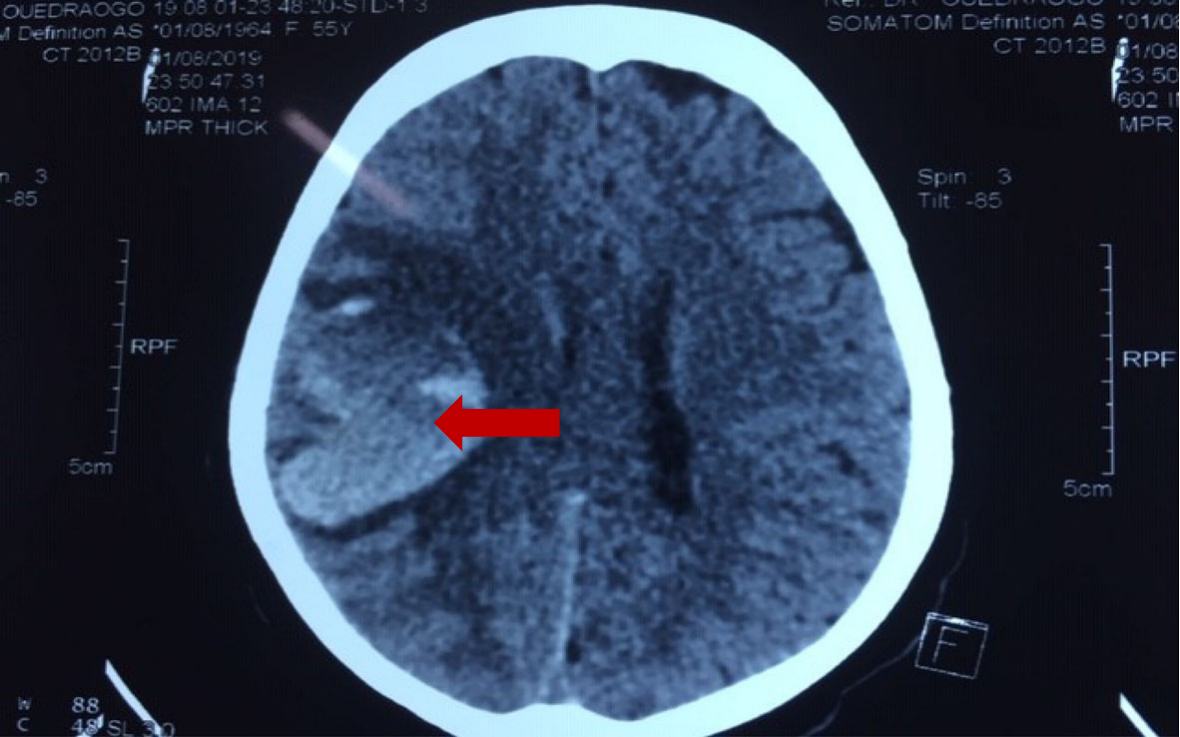
Cerebral Computed Tomography showing heterogeneous hyperdensity on internal capsule, lenticular nuclei in right hemisphere and cerebral edema, in a 55-year-old woman presenting with snake bite

A 55-year-old woman, residing in a rural area 35 km from Ouagadougou, presented on May 21, 2019 with a snake bite on the 5th right toe with associated bleeding at the gingival, abdominal, and left thigh. The patient did not have any past medical history, and the type of snake was unknown. The patient was treated by a traditional healer with scarifications. Twelve days following the initial presentation, she had sudden motor deficit of the left hemi-body preceded by headache, vomiting and fever. She was brought to a local hospital where she received etamsylate injectable, metronidazole injection, paracetamol injectable and ceftriaxone. Anti-snake venom (ASV) and anti-tetanus serum (ATS) were administered in this local hospital. Following admission in the neurology department, the patient presented with hyperthermia a 39 °C, blood pressure at 130/80 mmHg, tachycardia (124 beats per minute), an elevated respiratory rate at (30 cycles per mm), a scar at the level of the lesion, and hematoma of the left thigh. Neurological examination had showed flaccid left hemiplegia with a Medical Research Council score (MRC score) of grade 0 and a National Institute of Health Stroke. Cerebral Computed Tomography showed heterogeneous hyperdensity on internal capsule, lenticular nuclei in right hemisphere and cerebral edema (Fig. 1). The diagnosis of hemorrhagic stroke or venous thrombosis was discussed. The biological assessment found a D-dimer level at 7500 mg / l (15 time normal), a hyperleucocytosis predominantly neutrophilic (14,000 / mm 3), a moderate anemia (9.1 g / l), a negative thick drop, a negative blood culture, an elevated C Reactive protein (95 mg / l), a normal level of creatinine (73.umol / l), a normal level of urea (6 mmol / l), a normal level of Alamine transaminance (22 IU / l), and an elevated level of aspartate transaminance at 45 IU / l. The patient was treated with ceftriaxone 2 g / 24 h, metronidazole 500 mg three time per day. The patient received another dose of anti-snake venom during the 16 day hospitalization. The clinical course was marked by incomplete motor recovery with a MRC score of 0 on the upper limb and 2 on the lower limb.

### Case 2

**Fig. 2:**
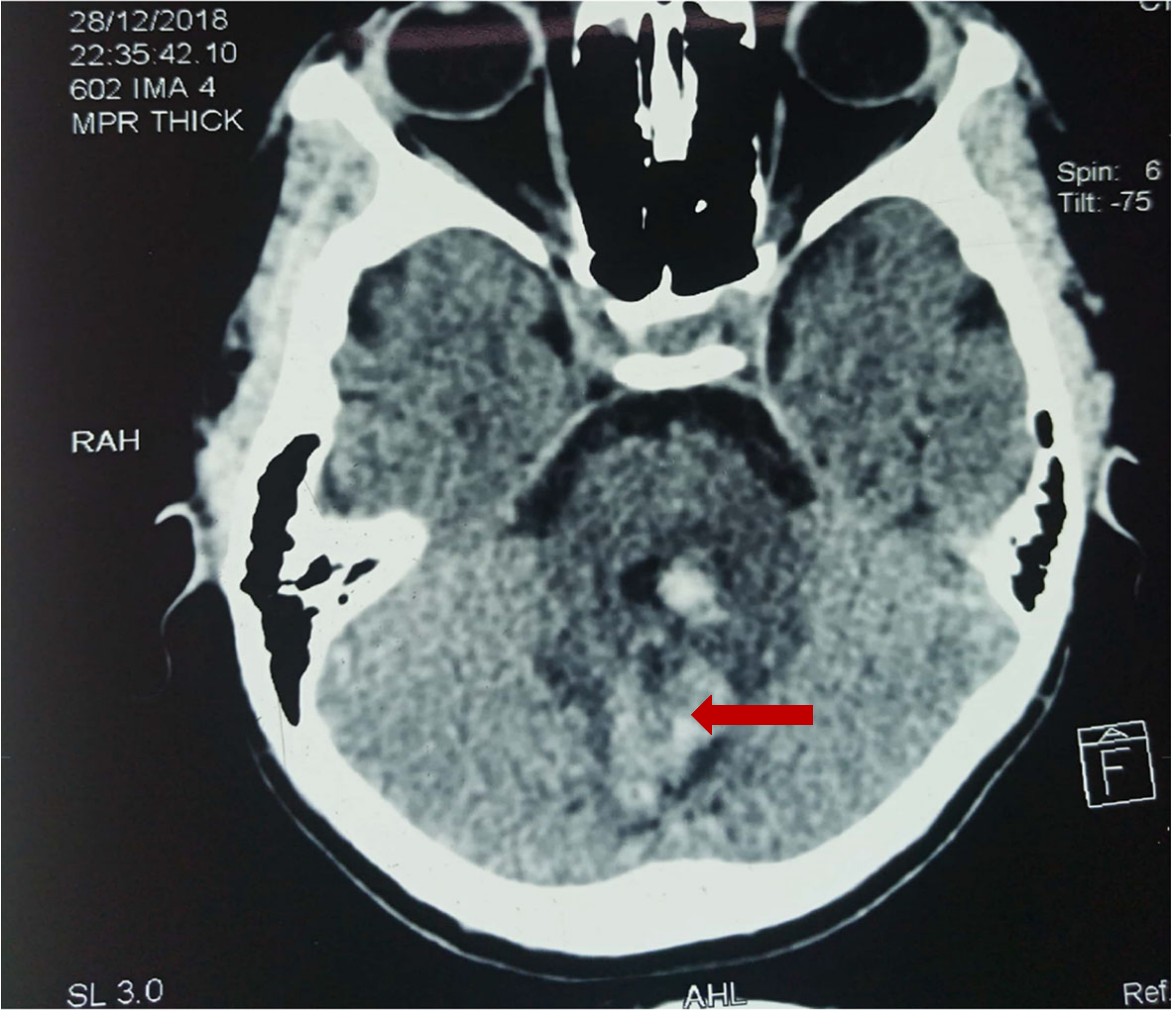
Cerebral CT showing spontaneous left cerebellar hemorrhage, in a 16-year-old male presenting with loss of consciousness and severe anemia following snake bite

A 16-year-old man, residing in a rural area 135 km from Ouagadougou, without past medical history, was referred to the Emergency Department for a snake bite of undetermined nature on December 12, 2018. The bite was located on the left arm which was completely distorted and painful. At the bite, there was a puntiform and ulcerative bleeding wound. The patient presented to a local medical center with significant bleeding at the insertion site of the venous tract, followed by a loss of consciousness. He had severe anemia (Hemoglobin at 5 g / dl) and received a blood transfusion of 2 blood bags. Following admission in the Infectious disease department, the patient endorsed acute headache without vomiting and constipation. Consciousness was unclear with obnibulation. There the patient had elevated blood pressure at 140/100 mmHg, normal body temperature, normal heart beat and a polypnea of 16 cycles per mm. Local examination revealed swelling of the left side of the upper limb and a lesion suggestive of the bite site. This envenomation was classified as grade 3 according to the Snakebite Severity Score. Biologically, white blood cells were at 7060 / mm3, C reactive protein at 8.39 mg / l, hemoglobin at 9.9 g / dl, VGM at 8.2, MCHC at 30.3, platelet count at 510000 / mm ^3^. Electrolyte test revealed a serum albumin level at 142.5 mmol / l, potassium at 4.24, calcium at 21.16. HSV serology was negative. After a few days following admission, the patient presented with sudden right hemiparesis (MRC: 0/5 at upper limb and 5/5 at lower limb), cerebellar ataxia and aphasia and then transferred later in the neurology department. The cerebral CT showed bilateral spontaneous frontal and left cerebellar hemorrhage **(**Fig. 2**).** The patient was treated with ceftriaxone and received a dose of antivenom serum during this hospitalization of 23 days. The clinical outcome was favorable and the patient recovered completely from this motor deficit.

### Case 3

**Fig. 3:**
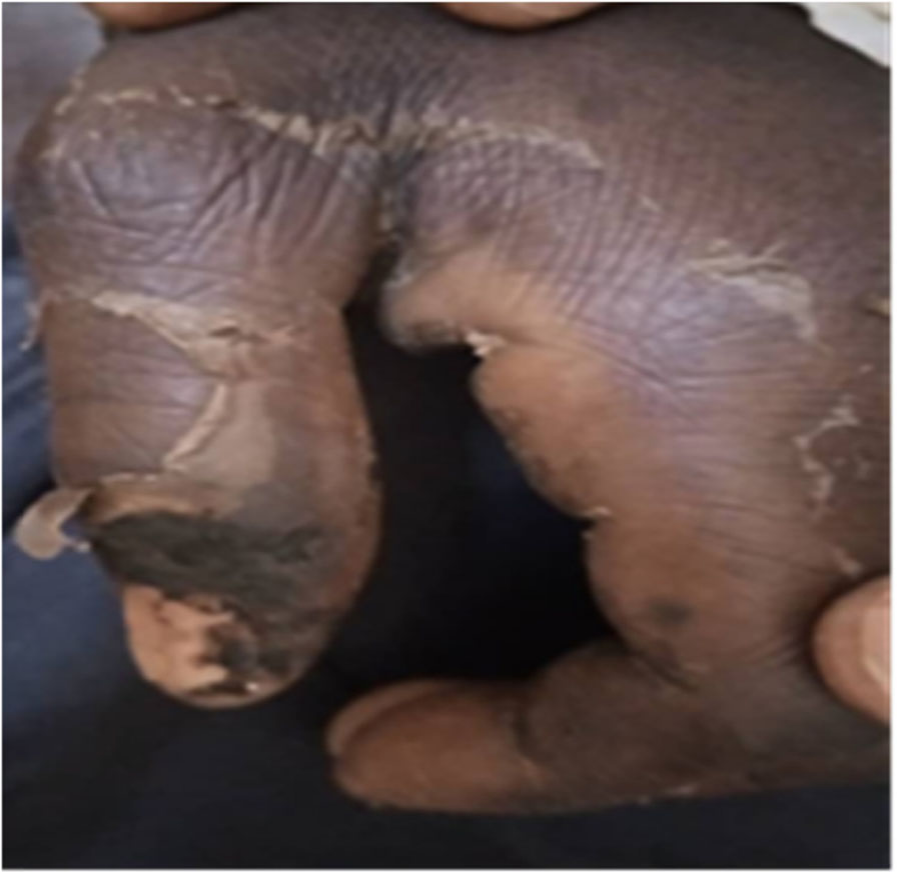
Image showing pronation stiffness of left upper limb at snake bite point, in a 30-year-old woman presenting with motor deficit, speech disorder, and consciousness disorder

**Fig. 4:**
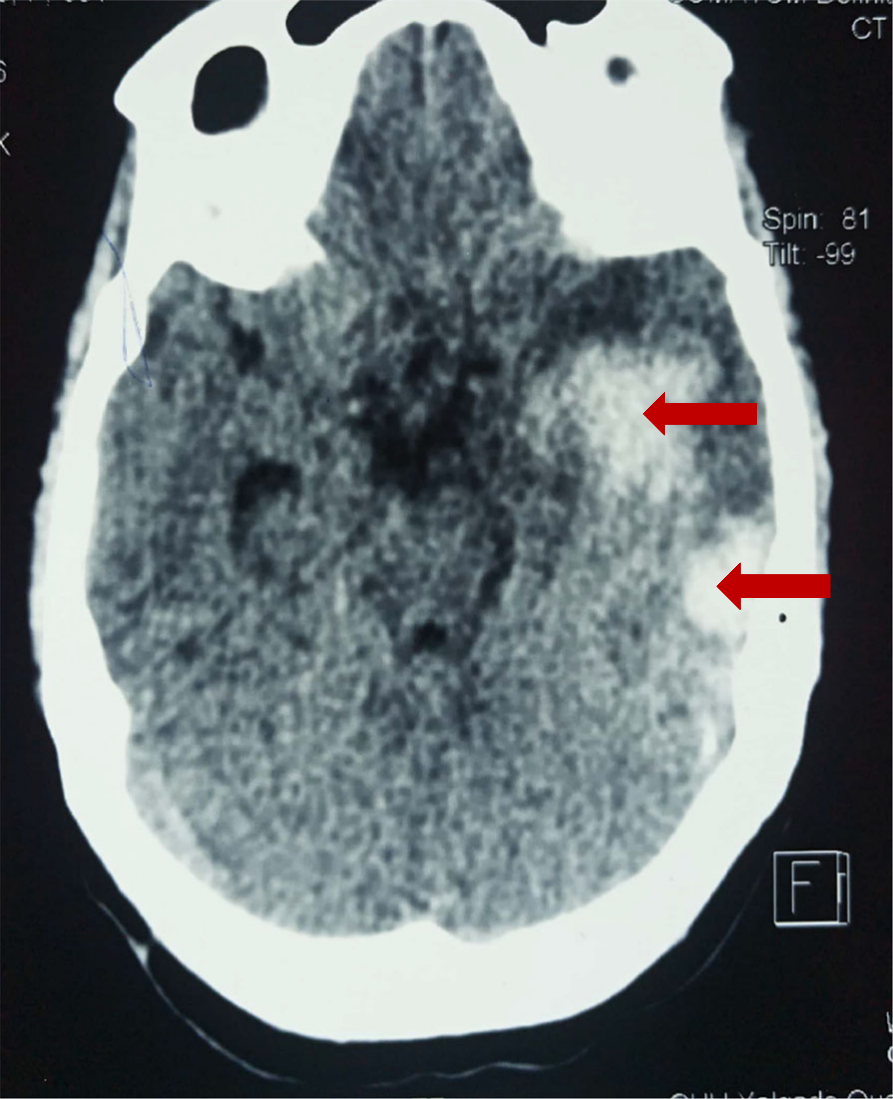
Brain CT showing right spontaneous temporal hematoma, in a 30-year-old woman presenting with motor deficit, speech disorder, and consciousness disorder

A 30-year-old woman living in a rural area 250 km from Ouagadougou, was admitted on August 19, 2019 in the Neurology Department of Yalgado Ouedraogo University Teaching Hospital or motor deficit on the right hemibody alongside a language disorder. She had no past medical history. The patient was bitten by a snake on the left arm with swelling and bleeding for 10 days, and was treated at home by a traditional healer for a week. After the onset of a sudden motor deficit and consciousness disorder, she was admitted for 4 days at a local hospital where she received antivenom serum, anti-tetanic vaccine, vitamin K, diazepam, and paracetamol. The snake was not identified by peripheral health workers. Despite adequate treatment, the state of consciousness worsened and the patient was transferred at the Neurology Department. General examination at the neurology department revealed elevated blood pressure at 140/90 mmHg, hyperthermia at 39 °C and hematuria with urine color “like coca cola”. Neurological examination had found Broca’s aphasia, left ptosis, Kerning and Brudzinkski signs alongside a 9 on the Glasgow Coma Scale. The dermatological examination showed a pronation stiffness of left upper limb (Fig. 3). Biological investigations had showed urea at 2.69, creatinine at 45.9, hemoglobin at 11 g / dl, white blood cell at 21,000 / mm 3; platelets at 28,000. Alanine transaminase was at 24 IU, aspartate transaminance at 12 IU. Prothrombin level was at 50%. Brain CT scan revealed two heterogeneous temporal hematoma measuring 15 ml and 1.7 ml, perihematomal oedema and left parietal dural hematoma (Fig. 4). The snake bite was classified Grade III according to the WHO classification system. The length of stay was 51 days. During hospitalization, she received blood transfusion of 2 red globular pellet pockets, anti-tetanus serum, anti-snake venom, antibiotic, analgesic and rehydration. The clinical course was favorable to full recovery of consciousness and motor deficit.

## Discussion

We have described the first three cases of hemorrhagic stroke after envenomation in the context of Burkina Faso. All the cases occurred in young patients (< 65 years of age) without vascular risk factors, following previous literature which suggests that only 2% of snake bite patients had a history of diabetes mellitus or hypertension [3]. All sexes are affected by hemorrhagic stroke [6, 9, 10] and all patients were agro-pastoralists, living in rural areas. People engaged in farming, hunting, fishing and other rural activities are at highest risk, mostly bitten on their limbs during work [11, 12]. Snake species belonging to six families including the most venomous, Elapidae and Viperidae, are found in Burkina Faso [13]. The type of snake was unknown to the patient in all the cases, as at least 75% of snakebite victims present to hospital with an unknown bite or with a bite from an unidentified species [14]. The identification of the snake responsible for hemorrhagic stroke but Elapidae has also been identified in a case of an 85-year-old woman [15]. Viperidae is the most common type responsible of hemorrhagic stroke but the Elapidae family was identified in a case of an 85-year-old woman [16]. The majority of our patients had consulted a traditional healer, as in the study of Somé in the southwest of Burkina Faso in 2002 [17]. This situation delayed the management of snake bite. The neurological manifestations observed in our patients were motor deficit, ptosis and coma. According to the literature, ptosis (85.7%), ophthalmoplegia (75%), and limb weakness (26.8%) are the most common neurological manifestations following snake bite [18]. Animal-derived antivenoms are considered the only specific therapy available for treating snakebite envenoming [19–21]. All the patients had received polyvalent anti-snake venom FAV-Afrique (FAV-A) before hospital admission, however administration delay is unknown. This polyvalent snake antivenom was elaborated by immunization of horses with venom from 10 different snake species among the most dangerous in Africa and belonging to Elapidae and Viperidea families. Treatment should be initiated as soon as possible but can be realized as long as the symptoms are present. Delayed treatment can be fatal, especially beyond six hours after the bite [22–25]. However, delayed administration of antivenom may be beneficial for patients with coagulopathies and local symptoms greater than six hours after envenomation [26]. Most of the patients received more than two doses of anti-snake venom during hospitalization time. FAV-A was associated with a low mortality rate of 1.8%; only 22% of patients required repeat antivenom doses [27]. Anti-tetanus serum was given to all the patients, in line with a study from Nigeria in which over 90% of snakebite victims received anti tetanus prophylaxis [28]. Tetanus prophylaxis is essential in snake bites because of the nature of the sites (puncture wounds) and the fact that snakes carry pathogenic organisms, including *clostridium tetani* as part of their normal oral microflora [29]. Tetanus could follow snake bite in inaccessible rural agriculture communities with inadequate health care provisions as well [30]. Antibiotic use was common following snake bite in the African context, as in Nigeria in which 67.4% of snake victims received antibiotics [28]. A short length stay was reported in snake bite victims, 5.7 ± 5.1 days (range < 1–23 days) in the study of Omogbait [28]. The long length of stay in our study (6 to 51 days) could be explained by the gravity of envenoming (grade 3 in two patients). In Africa, the lethality from snakebite is estimated to be between 2.8 and 11.6% whether or not antivenin is used [31]. Surprisingly, no mortality was recorded and all the patients had recovered from their neurological manifestations (conscience disorders, motor deficit, headache, high fever). This situation could be explained by several factors, in particular the absence of known defects in our patients, the early treatment of patients at local hospital and the absence of ventricular flooding on the brain scan.

### Limitations of cases studies

The case series had several limitations. The low number of patients included didn’t allow us to generalized the conclusions to the whole country. Some data such as patient management delays, dosages of snake bite venoms were missing. The evolution of biological data during the hospitalization of patients could not be described.

## Conclusion

Hemorrhagic stroke following snake bite remains rare in Burkina Faso. Neurological manifestations were severe with motor deficit and consciousness disorders. Antivenom, anti-tetanus serum, and antibiotics were utilized as front-line therapeutics. Clinical outcomes were favorable following treatment. This study should be extended to the most affected regions of Burkina Faso to determine risk factors.

## Data Availability

All the data and materials were available with the corresponding authors.
